# Retraction: miR-126a-3p induces proliferation, migration and invasion of trophoblast cells in preeclampsia-like rats through inhibiting a disintegrin and metalloprotease 9(ADAM9)

**DOI:** 10.1042/BSR-20191271_RET

**Published:** 2020-07-21

**Keywords:** miR-126a-3p, ADAM9, preeclampsia, trophoblast cells, biological characteristics

This article is being retracted from *Bioscience Reports* at the request of the authors following receipt of a notification from a reader, alerting the Journal to repeated features in Figures 3A, 3B, 9A and 10A.

The authors wish to retract the article. The Editor-in-Chief and Editorial Board agree with the retraction

